# Efficacy, Outcome, and Safety of Elderly Patients with Glioblastoma in the 5-ALA Era: Single Center Experience of More Than 10 Years

**DOI:** 10.3390/cancers13236119

**Published:** 2021-12-04

**Authors:** Barbara Kiesel, Lisa I. Wadiura, Mario Mischkulnig, Jessica Makolli, Veronika Sperl, Martin Borkovec, Julia Freund, Alexandra Lang, Matthias Millesi, Anna S. Berghoff, Julia Furtner, Adelheid Woehrer, Georg Widhalm

**Affiliations:** 1 Department of Neurosurgery, Medical University Vienna, 1090 Vienna, Austria; barbara.kiesel@meduniwien.ac.at (B.K.); lisa.wadiura@meduniwien.ac.at (L.I.W.); mario.mischkulnig@meduniwien.ac.at (M.M.); jessica.makolli@meduniwien.ac.at (J.M.); veronika.sperl@meduniwien.ac.at (V.S.); martin.borkovec@meduniwien.ac.at (M.B.); julia.freund@meduniwien.ac.at (J.F.); alexandra.lang@meduniwien.ac.at (A.L.); matthias.millesi@meduniwien.ac.at (M.M.); 2 Department of Medicine I, Clinical Division of Oncology, Medical University of Vienna, 1090 Vienna, Austria; anna.berghoff@meduniwien.ac.at; 3 Department of Biomedical Imaging and Image-Guided Therapy, Medical University Vienna, 1090 Vienna, Austria; Julia.Furtner@meduniwien.ac.at; 4 Department of Neurology, Institute of Neuropathology and Neurochemistry, Medical University Vienna, 1090 Vienna, Austria; adelheid.woehrer@meduniwien.ac.at

**Keywords:** elderly patients, glioblastoma, 5-aminolevulinic acid, resection, biopsy

## Abstract

**Simple Summary:**

In the next decades, the incidence of patients with glioblastoma (GBM) will markedly increase due to the growth of the elderly population. Despite the increasing incidence of GBM, elderly patients are frequently excluded from clinical studies and thus, only few data are available specifically focusing on the elderly population. In the current study, we aimed to investigate the efficacy, outcome, and safety of surgically-treated GBM including resections and biopsies in the 5-ALA era in a large elderly cohort of altogether 272 patients. Our data of this large elderly cohort demonstrate for the first time the clinical utility and safety of 5-ALA fluorescence in GBM for improved tumor visualization in both resections as well as biopsies. Therefore, we recommend the use of 5-ALA not only in resections, but also in open/stereotactic biopsies to optimize the neurosurgical management of elderly GBM patients.

**Abstract:**

Background: In the next decades, the incidence of patients with glioblastoma (GBM) will increase due to the growth of the elderly population. Fluorescence-guided resection using 5-aminolevulinic acid (5-ALA) is widely applied to achieve maximal safe resection of GBM and is identified as a novel intraoperative marker for diagnostic tissue during biopsies. However, detailed analyses of the use of 5-ALA in resections as well as biopsies in a large elderly cohort are still missing. The aim of this study was thus to investigate the efficacy, outcome, and safety of surgically- treated GBM in the 5-ALA era in a large elderly cohort. Methods: All GBM patients aged 65 years or older who underwent neurosurgical intervention between 2007 and 2019 were included. Data on 5-ALA application, intraoperative fluorescence status, and 5-ALA-related side effects were derived from our databank. In the case of resection, the tumor resectability and the extent of resection were determined. Potential prognostic parameters relevant for overall survival were analyzed. Results: 272 GBM patients with a median age of 71 years were included. Intraoperative 5-ALA fluorescence was applied in most neurosurgical procedures (*n* = 255/272, 88%) and visible fluorescence was detected in most cases (*n* = 252/255, 99%). In biopsies, 5-ALA was capable of visualizing tumor tissue by visible fluorescence in all but one case (*n* = 91/92, 99%). 5-ALA administration did not result in any severe side effects. Regarding patient outcome, smaller preoperative tumor volume (<22.75 cm^3^), gross total resection, single lesions, improved postoperative neurological status, and concomitant radio-chemotherapy showed a significantly longer overall survival. Conclusions: Our data of this large elderly cohort demonstrate the clinical utility and safety of 5-ALA fluorescence in GBM for improved tumor visualization in both resections as well as biopsies. Therefore, we recommend the use of 5-ALA not only in resections, but also in open/stereotactic biopsies to optimize the neurosurgical management of elderly GBM patients.

## 1. Introduction

Glioblastoma (GBM) is the most common primary malignant brain tumor with very poor prognosis and predominance in the elderly population [1]. The incidence of elderly GBM patients aged ≥ 65 years is 2.6 times higher than in younger patients [2]. At present, approximately half of GBM patients are aged ≥ 65 years at the time of diagnosis [3,4]. The anticipated higher life expectancy over the next decades represents a crucial factor for the increasing incidence of elderly GBM patients [5]. In 2030, the age group ≥ 65 years is expected to account for two thirds of all GBM patients according to epidemiological studies [3,4]. Despite the increasing incidence of GBM cases, elderly patients are frequently excluded from clinical studies or inadequately represented and thus, only few data are available specifically focusing on the elderly population [2]. Thus, optimization of the management of GBM patients within this elderly population is of particular importance [5].

The current treatment in GBM constitutes a multimodal approach including maximal safe resection or biopsy followed by postoperative concomitant radio-chemotherapy [1,6]. In routine clinical practice, however, incomplete resections of the contrast-enhancing tumor, as well as acquisition of non-diagnostic tissue samples during biopsies of suspected GBM, are not uncommon due to insufficient intraoperative tumor visualization [7]. Currently, improved intraoperative tumor visualization with 5-aminolevulinic acid (5-ALA) fluorescence is frequently applied in the neurosurgical field [8,9,10,11,12,13,14]. Generally, 5-ALA is well-tolerated and associated with a negligible rate of side effects. [8] At present, the main indication of 5-ALA is fluorescence-guided resection of GBM resulting in a significantly higher rate of complete resections and longer progression-free survival compared to conventional white-light surgery [8]. Therefore, this innovative approach is the current standard for optimal GBM resection at many centers worldwide [8,15]. Additionally, we identified 5-ALA fluorescence as a novel marker for diagnostic tumor tissue during stereotactic biopsies, which is useful especially in high-grade gliomas (HGG) [9,10,11,13]. The significance and safety of 5-ALA fluorescence for both resections as well as biopsies of GBM have not been systematically investigated in detail in an elderly cohort thus far. However, these data would be of major importance due to the expected distinct increase of elderly GBM patients in the next decades [2,3,4].

At our institution, we have access to a large cohort of elderly patients aged ≥65 years with resection or biopsy of a GBM in the 5-ALA era. Since biopsy is a commonly performed procedure in this elderly age group, we also included these standard neurosurgical procedures aside from resections of GBM in this study. The aim of this study was thus to retrospectively investigate the efficacy, outcome, and safety of this surgically-treated elderly cohort.

## 2. Materials and Methods

In this retrospective study, all patients aged ≥ 65 years with neurosurgical interventions (resection or biopsy) of a World Health Organization (WHO) grade IV glioma since the introduction of 5-ALA fluorescence procedures at the Department of Neurosurgery, Medical University Vienna (MUV) were included. According to our inclusion criteria, we included patients with a histopathologically confirmed, newly diagnosed, and recurrent WHO grade IV glioma between 2007 and 2019. This study was approved by the local ethics committee of the MUV (EK 419/2008).

### 2.1. Preoperative Imaging

Generally, patients with a suspected GBM received preoperative magnetic resonance imaging (MRI) with contrast-media application within 2 weeks prior to surgery. For integration into neuronavigation, contrast-enhanced T1-weighted images and additional sequences, such as diffusion tensor imaging (DTI) and/or functional MRI (fMRI), were conducted depending on tumor localization and type of surgery. The pattern of contrast-enhancement (CE) on T1-weighted images was classified by a neuroradiologist (J.F.) as ring-like, nodular, focal, patchy/faint, or none as described previously [10,16]. Additionally, tumor eloquence was determined using the criteria defined by Chang et al. [17]. Moreover, all tumors were classified as single lesion, multifocal lesion (multiple contrast-enhancing lesions with a distinct connection on FLAIR/T2-weighted images), or multicentric lesion (lack of a distinct connection on FLAIR/T2-weighted images between multiple contrast-enhancing lesions) [18]. Furthermore, preoperative tumor volume was measured according to the contrast-enhancing lesion using the open-source segmentation software ITK-SNAP (version 3.6.0, http://www.itksnap.org, accessed on 3 December 2021). Finally, all tumors undergoing resection were classified as completely resectable or not completely resectable by 2 experienced neurosurgeons (G.W., B.K.) based on preoperative MRI. These 2 neurosurgeons were blinded to the achieved extent of resection (EOR) according to postoperative MRI.

### 2.2. 5-ALA in Neurosurgical Procedures

The type of surgery (resection vs. open/stereotactic biopsy) was individually determined for each patient mainly dependent on tumor localization/eloquence, tumor expansion, and presence of comorbidities. In all patients, we used neuronavigation for intraoperative guidance. We screened our databank to determine whether 5-ALA was applied during surgery and if potential 5-ALA-related side effects occurred. In patients with fluorescence application, a standard dosage of 5-ALA (20 mg/kg bodyweight) was administered approximately 3 to 4 hours prior to anesthesia. For intraoperative visualization of fluorescence, a modified surgical microscope was used [9,12,16]. All patients were protected from strong light sources for at least 24 h after 5-ALA administration to minimize the potential risk of phototoxic reactions.

#### 2.2.1. Tumor Resections

In the case of resection, intraoperative monitoring, navigation with DTI/fMRI, and/or awake surgery was usually applied depending on tumor localization/eloquence to achieve the surgical goal of a maximal safe tumor removal. In the case of fluorescence-guided resection, the surgeon repeatedly switched to violet-blue excitation light to visualize potential fluorescence. The maximum visible fluorescence status (strong, vague, or no fluorescence) of each tumor was derived from our 5-ALA databank. Additionally, data on the presence of visible fluorescence within the resection cavity at the end of surgery were collected from our databank.

#### 2.2.2. Open/Stereotactic Biopsies

In open biopsies, navigation-guided sampling from a region of contrast-enhancement was conducted for histopathological analysis following a small craniotomy and the fluorescence status (strong, vague, or no fluorescence) was checked in cases with 5-ALA administration. In stereotactic biopsies, we used a navigated-biopsy-arm for tissue collection from the contrast-enhancing tumor region as described previously [9,11,13,14]. In the case of 5-ALA administration, the collected samples were investigated for potential visible fluorescence (strong, vague, or no fluorescence) directly in the operating room using a sterile microscope [9,11,13]. Data on the maximum fluorescence status (strong, vague, or no fluorescence) of open/stereotactic biopsies were collected from our databank.

### 2.3. Postoperative Imaging

Routinely, a computerized tomography (CT) was performed 1 day after resection/biopsy to detect postoperative complications such as hemorrhages and/or ischemia. For this study, we screened postoperative CT and clinical data for occurrence of symptomatic hemorrhages. In cases of resection, we usually conducted an additional postoperative MRI preferably within 72 h after surgery to determine the EOR: (1) gross total resection (GTR) in cases of ≥95% resection of the contrast-enhancing tumor, and (2) subtotal resection (STR) in cases of <95% removal of the contrast-enhancing tumor [19]. Based on postoperative MRI, we measured the volume of a potential contrast-enhancing residual tumor.

### 2.4. Histology, Clinical Data, Postoperative Neurological Outcome, and Adjuvant Treatments

Histopathological diagnosis was established according to the current WHO classification at the time of diagnosis [1,20]. According to our inclusion criteria, we only included patients with histopathologically confirmed WHO grade IV gliomas. Routinely, patients stayed at the neurosurgical department approximately 5 to 10 days after complication-free-surgery. Furthermore, patient medical records were screened for clinical data, such as the preoperative Karnofsky index, pre- and postoperative neurological status, postoperative ECOG performance status, known comorbidities, number of re-do surgeries, postoperative adjuvant treatments, number of patients that were fit for further postoperative treatment, and overall survival. Regarding the neurological status, data were obtained before surgery, at time of discharge and at a follow-up visit approximately 3 months after surgery. The neurological status was classified as improved, stable, or worse at time of discharge from hospital, and a neurological deficit was considered temporary in the case of recovery at the 3-month follow-up visit. In order to assess the known comorbidities in relation to the patient’s age, we calculated the Charlson Comorbidity Index (CCI) for each patient. The CCI is generally used to predict the 1-year mortality risk according to specific comorbidities and patient’s age (see Table S1). Finally, the modality of postoperative treatments was routinely discussed in an interdisciplinary tumor board in each patient and arranged accordingly.

### 2.5. Statistical Analysis

All analyses were executed using R 4.1.0 (R Core Team 2021, R: A language and environment for statistical computing https://www.R-project.org/, accessed on 3 December 2021) and SPSS 26.0.0.0 (IBM SPSS Statistics, IBM, Armonk, New York, US). Differences in numerical or ordinal variables between groups were examined using the Wilcoxon rank-sum test or Kruskal-Wallis test. Univariate influence on overall survival was assessed with the logrank test. Results were statistically significant at a two-sided significance level *p* < 0.05.

## 3. Results

Our cohort comprised 272 elderly patients (≥65 years) with surgery of a newly diagnosed or recurrent WHO grade IV glioma at our department since the introduction of 5-ALA fluorescence procedures. Altogether, 289 neurosurgical procedures including 182 (63%) resections and 107 (37%) biopsies were conducted. The median age of our elderly cohort was 71 years (range 65–88). According to histopathological analysis, a GBM was diagnosed in most cases (269 of 272 patients, 99%) and a gliosarcoma in the remaining three (1%) cases. For further details see Table 1.

### 3.1. Tumor Characteristics

The study cohort constituted 264 (91%) newly diagnosed and 25 (9%) recurrent tumors. The most common tumor localization was the temporal lobe (*n* = 86, 30%), followed by the frontal (*n* = 67, 23%) and parietal lobe (*n* = 38, 13%). Eloquent tumor localization was present in 163 (56%) cases. In most cases a single lesion (*n* = 217, 75%) was noted on preoperative MRI, whereas multifocal or multicentric lesions were observed in 72 (25%) cases. CE on T1-weighted images was present in all tumors with available preoperative MRI (*n* = 285). In detail, ring-like CE was observed in 254 (88%) cases and nodular CE in 23 (8%) cases. The mean preoperative volume of the contrast-enhancing tumor was 26.8 cm^3^ (range 0.4–121 cm^3^). Further details are provided in Table 2.

### 3.2. Preoperative Comorbidities, Performance Status and Neurological Symptoms

**Figure 1:**
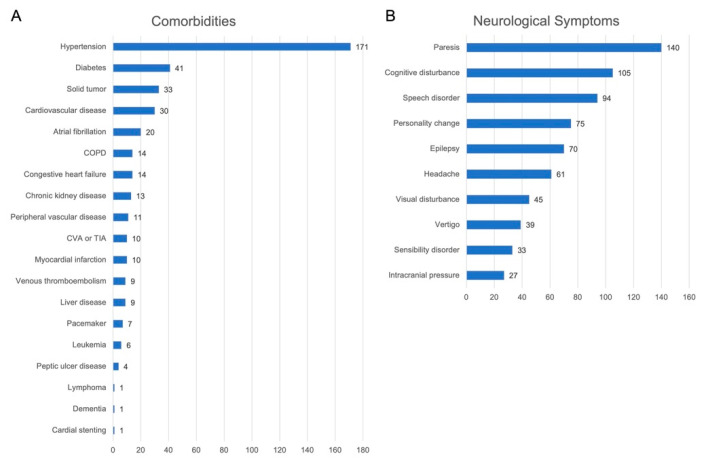
Detailed list of known comorbidities and neurological symptoms of the study cohort. (**A**) Detailed list of known comorbidities of our elderly cohort. Hypertension represented the most common comorbidity followed by diabetes, solid tumors, and cardiovascular disease. (**B**) Detailed list of neurological symptoms present in our elderly cohort. Paresis represented the most common neurological symptom in our elderly cohort, followed by cognitive disturbance, speech disorder, and personality changes.

At the time of hospital admission, 212 (78%) of 272 patients had one or more known comorbidities with a median CCI of 4 points (range 2–11). In detail, hypertension represented the most common comorbidity (*n* = 171, 63%) followed by diabetes (*n* = 41, 15%). A detailed list of known comorbidities in our patient cohort is provided in Figure 1A. Additionally, the median preoperative Karnofsky index was 70% (range 20–100%), whereas male patients had a significantly higher preoperative Karnofsky index compared to female patients (80% vs. 70%; *p* = 0.0261). Regarding neurological function, motor deficit was the most common neurological symptom in our cohort (*n* = 134, 49%). For further details see Figure 1B.

### 3.3. Neurosurgical Procedures

In most tumors a resection (*n* = 182, 63%) was performed. In the remaining cases either a stereotactic biopsy (*n* = 84, 29%) or open biopsy (*n* = 23, 8%) was conducted. Patients with resections were significantly younger compared to patients with open/stereotactic biopsies (69 years vs. 72 years; *p* < 0.001). Further, a significantly higher preoperative tumor volume was noted in cases with resections (mean 30.3 cm^3^) compared to stereotactic/open biopsies (mean 21.0 cm^3^; *p* < 0.001).

### 3.4. Intraoperative Application of 5-ALA Fluorescence

Intraoperative 5-ALA fluorescence was applied in most cases (*n* = 255, 88%). In contrast, 34 (12%) surgeries were performed without 5-ALA administration. The most common reasons for lack of 5-ALA administration were a suspected other tumor entity (*n* = 9, 26%), emergency surgery (*n* = 4, 12%), inadequate administration (*n* = 4, 12%), and elevated liver enzymes (*n* = 2, 6%). Further details are given in Table S2.

#### 3.4.1. Tumor Resections with Assistance of 5-ALA

**Figure 2:**
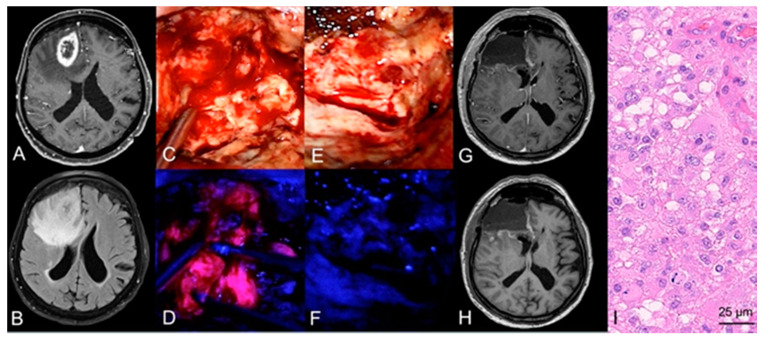
Case illustration of a fluorescence-guided resection of a GBM. (**A**) Preoperative MRI demonstrates a lesion in the right frontal lobe with ring-like contrast-enhancement on T1-weighted sequences and hyperintensity on (**B**) FLAIR sequences in a 72-year-old male patient. (**C**) During tumor resection under white-light microscopy, (**D**) the neurosurgeon repeatedly switches to violet-blue excitation light to visualize tumor tissue with assistance of visible 5-ALA fluorescence. (**E**) At the end of surgery, the performing neurosurgeon investigates the resection cavity for potential residual tumor tissue and (**F**) does not detect any residual 5-ALA visible fluorescence. (**G**,**H**) After resection of the tumor, postoperative MRI including T1-weighted sequences with and without contrast-media show a complete resection of the contrast-enhancing tumor. (**I**) Histopathological analysis reveals a glioblastoma WHO grade IV.

In the case of resection, 5-ALA fluorescence-guided surgery was conducted in 163 (90%) cases. Of these 163 cases, 5-ALA was able to detect tumor tissue during surgery by visible fluorescence in 161 (99%) cases. In detail, strong 5-ALA fluorescence was found as maximal fluorescence level in 153 (94%) cases, vague fluorescence in eight (5%), and no visible fluorescence in two (1%) cases. At the end of resection, a complete removal of visible fluorescence was achieved in 50 (31%) cases and in 111 (68%) cases residual fluorescence was present. In the remaining two cases, no information on residual fluorescence at the end of surgery was available. An illustrative case is provided in Figure 2.

#### 3.4.2. Tumor Biopsies with Assistance of 5-ALA

**Figure 3:**
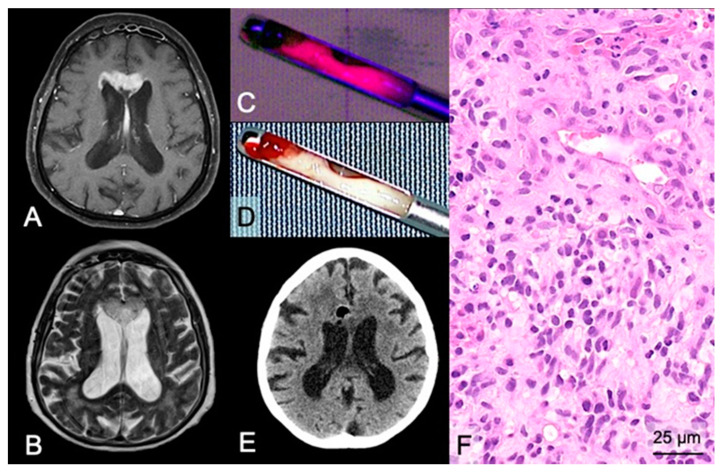
Case illustration of a stereotactic biopsy using 5-ALA fluorescence in a suspected GBM. (**A**) Preoperative MRI demonstrates a lesion in the midline/corpus callosum in the frontal lobes with contrast-enhancement on T1-weighted sequences and (**B**) hyperintensity on FLAIR sequences of a 70-year-old female patient. (**D**) During stereotactic biopsy, the neurosurgeon checks the collected tissue sample of the target region with significant contrast-enhancement under the sterile neurosurgical microscope using white-light and (**C**) violet-blue excitation light to investigate the fluorescence status revealing strong fluorescence. (**E**) Postoperative CT shows no hemorrhage in the biopsy area, and the air bubble indicates the correct biopsy site of the obtained tissue samples. (**F**) Histopathological analysis reveals a glioblastoma WHO grade IV.

In the case of biopsy, 5-ALA was used for intraoperative visualization of diagnostic tumor tissue in 92 (86%) stereotactic/open biopsies. Of these, 5-ALA fluorescence was capable of visualizing tumor tissue in 91 cases (99%). In detail, 83 (90%) cases showed strong fluorescence and 8 (9%) cases vague fluorescence as maximal fluorescence level. An illustrative case is provided in Figure 3. It is of note that the only case (open biopsy) with an absence of visible fluorescence showed massive necrotic/hemorrhagic features with only rare tumor cell infiltration of a GBM.

### 3.5. Extent of Resection

In order to clarify if there is a difference in EOR between completely and not completely resectable tumors, we investigated the resectability based on preoperative imaging. In total, 103 (56%) of 182 surgically-treated lesions were classified as completely resectable according to preoperative MRI. A clear statement on EOR was possible in 94 (91%) completely resectable lesions with available postoperative MRI. In the remaining nine cases (9%), a clear statement on EOR was not possible due to ambiguous postoperative MRI findings. In 84 (89%) of these 94 completely resectable tumors, GTR was achieved according to postoperative imaging. In two patients with STR with an unexpected, resectable residual tumor, a re-do surgery was conducted within the same hospital. A significantly higher rate of GTR was present in lesions classified as completely resectable (*p* < 0.001) and with non-eloquent tumor localization (*p* < 0.001). Additionally, we found a significantly higher median preoperative tumor volume in lesions with STR (81.4 cm^3^) compared to GTR (55.6 cm^3^; *p* < 0.001). Regarding the presence of visible fluorescence at the end of resection, GTR was significantly more common in tumors with complete removal of visible fluorescence compared to residual fluorescence (*p* < 0.001).

### 3.6. Safety: 5-ALA Related Side Effects, Perioperative Complications, and Postoperative Course

In this study, 5-ALA administration did not result in any severe side effects and was well-tolerated by our elderly cohort. We did not observe any clinically relevant severe side effects/symptoms related to 5-ALA administration also regarding kidney function or unexpected reactions. According to postoperative CT and clinical data, a symptomatic postsurgical hemorrhage was observed in 11 (4%) cases. Of these, one patient showed improvement of neurological deficits at the 3-month follow-up visit. In our cohort, an improved or stable neurological status at time of discharge from hospital was observed in 153 (84%) of 182 patients after resections, 77 (92%) of 84 patients after stereotactic biopsies, and 20 (87%) of 23 patients after open biopsies. A postoperative neurological deterioration was found in 29 (16%) of 182 resections and 10 (9%) of 107 biopsies. The rate of postoperative neurological deterioration did not differ significantly between resections and biopsies in our elderly cohort (*p* = 0.153). Of the 39 patients with postoperative neurological deterioration, six (15%) showed a temporary deficit which recovered at the 3-month follow-up visit. Of the patients with GTR (*n* = 108), postoperative neurological deterioration was observed in 15% (16 of 108) of cases at time of discharge. Regarding EOR, we did not find a significant difference in neurological deterioration in patients with GTR (*n* = 16 of 108, 15%) compared to STR (*n* = 7 of 55, 13%; *p* = 0.458). Recovery of a postoperative neurological deterioration after GTR was observed in three patients at the 3-month follow-up in our elderly cohort. In the remaining cases, no improvement of the neurological deficit was found at the 3-month follow-up in 11 patients, one patient died, and in one patient no follow-up was available. Finally, we also correlated the preoperative tumor volume with the neurological status at the 3-month follow-up. However, we did not observe a significant difference in neurological deterioration between patients with a smaller (<22.75 cm^3^) and larger (≥22.75 cm^3^) tumor volume at the 3-month follow-up visit (*p* = 0.378).

### 3.7. Postoperative Treatment

Altogether, 222 (82%) of 272 patients with newly diagnosed and recurrent tumors were fit for further postoperative treatment (radio- and/or chemotherapy). We did not find a statistically significant difference in the rate of patients fit for further postoperative treatment between GTR and STR (*p* = 0.289). In newly diagnosed WHO grade IV glioma, 201 (76%) of 264 patients received postoperative adjuvant treatment. In 46 (17%) cases, poor general/neurological condition, death, or rejection of further treatment impeded any adjuvant therapy and 17 (7%) patients were lost to follow-up. We compared the rate of patients in which no postoperative chemo-/radiotherapy was initiated between patients with GTR and STR. According to our data, we did not find a statistically significant difference in patients in which no postoperative chemo-/radiotherapy was initiated between cases with GTR and STR (*p* = 0.207). In 88 (44%) patients, concomitant radio-chemotherapy according to the “Stupp” scheme was performed. Furthermore, 80 (40%) patients were treated according to an “elderly” scheme including the “Perry” (radiotherapy with 40.05 Gy and temozolomide) and “Nordic glioma” (radiotherapy with 34 Gy and temozolomide) protocol. The remaining 33 (16%) patients received a variety of different treatment schemes. For further details see Table S3.

### 3.8. Impact of Specific Parameters on Overall Survival

**Figure 4:**
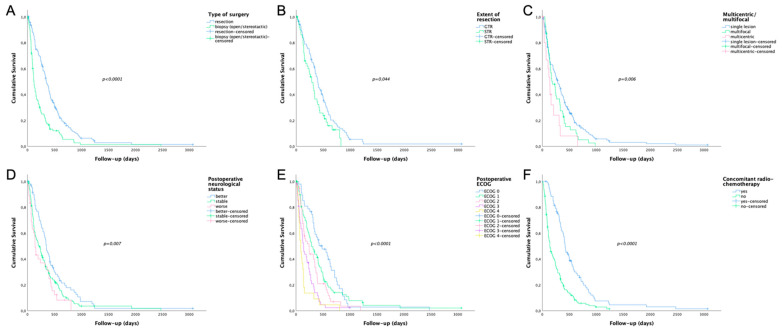
Survival plots of specific parameters significantly influencing overall survival. (**A**) Patients with tumor resection (median 348 days) had a significantly longer median overall survival compared to patients with open/stereotactic biopsies (median 121 days) (**B**) Regarding extent of resection, the median overall survival was significantly longer in patients with gross total resection (median 399 days) compared to subtotal resection (median 286 days). (**C**) Patients with a single lesion (median 285 days) had a significantly longer median overall survival compared to multifocal (median 188 days) or multicentric lesions (median 139 days). (**D**) In case of postoperative neurological status, patients with improved neurological status (median 350 days) had a significantly longer median overall survival compared to patients with stable (median 225 days) or worse neurological status (median 139 days). (**E**) The ECOG score was positively associated with overall survival (*p* < 0.001) revealing the longest median overall survival for patients with ECOG 0 (median 482 days). (**F**) Patients treated according to the “Stupp” protocol (median 421 days) showed a significantly longer median overall survival compared to other treatment protocols (median 139 days).

Our study cohort had a median overall survival of 8 months (median 251 days). Regarding preoperative tumor volume, patients with a tumor volume smaller than 22.75 cm^3^ had a significantly longer median overall survival (*p* = 0.003). Furthermore, we found a significantly longer median overall survival in patients with resections (median 348 days) compared to patients with open/stereotactic biopsies (median 121 days; *p* < 0.001, see Figure 4A). Regarding EOR, the median overall survival was significantly longer in patients with GTR (median 399 days) compared to STR (median 286 days; *p* = 0.044, see Figure 4B). Additionally, patients with a single lesion (median 285 days) had a significantly longer median overall survival compared to multifocal (median 188 days) or multicentric lesions (median 139 days, *p* = 0.006, see Figure 4C). Moreover, patients with improved neurological status after surgery (median 350 days) had a significantly longer median overall survival compared to patients with stable (median 225 days) or worse neurological status (median 139 days; *p* = 0.007, see Figure 4D). The ECOG score was positively associated with overall survival (*p* < 0.001) revealing the longest median overall survival for patients with ECOG 0 (median 482 days). In contrast, patients with ECOG 4 had a median overall survival of 87 days (see Figure 4E). Finally, compared to other treatment protocols patients treated according to the “Stupp” protocol (median 421 days) showed a significantly longer median overall survival (median 139 days; *p* < 0.0001, see Figure 4F). In contrast, age, gender, and Charlson Comorbidity Score had no significant impact on overall survival.

## 4. Discussion

Neurosurgical resection or biopsy is generally the initial step in the management of GBM [1,8,21]. In resections, maximal safe removal of the contrast-enhancing tumor with preservation of neurological function is the surgical goal [7,21]. In order to improve the EOR, 5-ALA fluorescence-guided resection is presently frequently applied in GBM patients since its approval in the European Union in 2007 and the United States in 2017 [8,15]. In specific GBM localizations such as deep-seated and/or bihemispheric tumors, an open or stereotactic needle biopsy is frequently conducted to collect tumor tissue for histopathological diagnosis [21]. Further, certain internal comorbidities present especially in elderly patients often favor a biopsy of a suspected GBM over an aggressive resection [22]. Recently, we identified 5-ALA fluorescence as a novel marker for diagnostic tumor tissue also in stereotactic biopsies such as HGG or lymphomas [9,10,11,13]. Although epidemiological studies expect a distinct increase of the population aged ≥65 years by 238% in 2050 [2], resulting in a notable rise of elderly GBM patients in the future, systematical data on the optimal surgical management of GBM patients in this age group in the 5-ALA era are missing.

### 4.1. Present Study

At our institution, we have access to a large patient cohort with resection or biopsy of a GBM since the implementation of the 5-ALA technique. Thus, we designed the present study and retrospectively investigated the efficacy, outcome, and safety in a large cohort only including elderly patients (≥65 years; median age 71 years). In order to focus not only on a very specific group of patients, for example patients with the goal of GTR, we included in our study a consecutive series of neurosurgically-treated elderly GBM patients and thus, also included biopsies aside from resections to provide a representative cohort with typically performed types of surgeries in this specific age group. Therefore, different extents of resections (GTR and STR) as well as biopsies were available for specific analyses in our elderly cohort.

Overall, 5-ALA was applied for improved intraoperative GBM visualization in most of the 272 patients (88%). This high rate of 5-ALA cases in this elderly population is explained by the fact that fluorescence was not only applied for the main indication of GBM resection, but also during biopsies within previous clinical studies [11,14].

### 4.2. Tumor Resections with 5-ALA

In our elderly cohort, visible 5-ALA fluorescence was found during resection in the vast majority of GBM (99%). Thus, we found similar high fluorescence rates compared to previous studies in younger cohorts. In this sense, visible 5-ALA fluorescence was found in a series of GBM patients with a markedly younger median age of 60 years in all 77 cases [12]. Similarly, Coburger et al. reported visible 5-ALA fluorescence in all 33 GBM in a cohort with a mean age of 57 years [23]. Further studies described visible 5-ALA fluorescence in HGG in 90% to 100% of cases in younger cohorts with a median age ranging between 49 and 60 years [24,25,26]. To clarify if there is a difference in EOR between completely and not completely resectable GBM, we investigated the resectability based on preoperative imaging. By using fluorescence-guided resection in most cases, we found a very high rate of GTR (89%) in completely resectable tumors in our elderly cohort. In previous studies in younger cohorts with a median age ranging between 57 and 60 years, GTR rates between 54% and 83% were reported [27,28,29,30]. It is of note that we observed a significantly higher rate of complete resections of the contrast-enhancing tumor in cases with complete removal of the entire visible fluorescence. Based on our data, intraoperative visualization of GBM tissue with 5-ALA fluorescence is thus a powerful technique during resection resulting in a high rate of GTR also in elderly patients.

Interestingly, we found a significantly higher preoperative tumor volume in cases with STR. Although 5-ALA is unaffected by brain shift, the likelihood of STR is expected to be higher in larger tumors due to extensive resection cavities with an increased risk of unrecognized residual tumor tissue. To overcome this limitation, the combination of 5-ALA fluorescence and intraoperative MRI might be a promising approach. In this sense, a recent study showed GTR in 100% of GBM using the combination of 5-ALA with intraoperative MRI [23]. However, intraoperative MRI is not widely available, and the significant prolongation of surgical/anesthesia time might limit its use in elderly patients.

Furthermore, we investigated if GTR achieved mostly by 5-ALA fluorescence-guided resection results in a higher rate of neurological deficits in our elderly cohort. According to our data, postoperative neurological deterioration was noted in our elderly cohort in 16 (15%) of 108 GBM in which GTR was performed. Mirza et al. analyzed in a recent study altogether 253 GBM patients with 5-ALA fluorescence-guided surgery [31]. This study cohort was markedly younger than patients included in our study (median: 57 years vs. 71 years) [31]. This study reported a new temporary neurological focal deficit in patients with GTR in 45% of cases [31]. The study performed by Mirza et al. found that a new temporary neurological focal deficit was less common in patients with STR compared to cases with GTR (18% vs. 45%) [31]. It is of note that we did not find a significant difference in neurological deterioration in patients with GTR (15%) compared to STR (13%) in our elderly cohort.

Finally, Ferroli P et al. investigated different factors for postoperative outcome after brain tumor resection in elderly patients and identified the occurrence of postoperative complications as important factor for postoperative worsening at the 3-month follow-up [32]. Similarly, we also found that only one of the 11 patients with a symptomatic postsurgical hemorrhage showed improvement of neurological deficits at the 3-month follow-up visit.

### 4.3. Tumor Biopsies with 5-ALA

In open/stereotactic biopsies, 5-ALA was capable of visualizing tumor tissue by visible fluorescence in all but one case (99%) directly in the operating room. In a previous study, we found visible 5-ALA fluorescence in all 25 GBM in a cohort of different brain tumors with a younger median age of 62 years [9]. Since we found a positive predictive value of 100% of strong fluorescence for diagnostic tumor tissue, we modified the biopsy strategy at our institution and we thus terminate the procedure in cases with strong 5-ALA fluorescence without waiting for intraoperative histopathology (frozen section) [9]. By this approach, we were able to significantly reduce the duration of the neurosurgical needle biopsies (41 vs. 77 min) which may be essential especially in elderly patients [11]. This approach is thus of special relevance in elderly patients representing a vulnerable cohort with a higher rate of surgical/anesthesiologic complications [33].

### 4.4. Specific Prognostic Factors in Elderly Patients

We also analyzed potential prognostic factors in our elderly cohort. Interestingly, elderly patients with a tumor volume smaller than 22.75 cm^3^ had a significantly longer overall survival. Accordingly, Xue et al. postulated the preoperative tumor volume as an important prognostic factor in HGG [34]. However, other studies did not find a significant impact of preoperative tumor volume on overall survival in HGG [35,36]. Further, elderly GBM patients with resections showed a significantly longer overall survival compared to biopsies in our study. This survival benefit of GBM patients undergoing resection is in accordance with the literature in younger cohorts [21,37]. Thus, resection should not be withheld from elderly GBM patients, and their management should change towards a personalized treatment in consideration of the individual patient’s general health. Moreover, we found a significantly longer survival in elderly patients in which GTR was achieved in our study. Although various studies identified maximal safe resection as a crucial prognostic factor in GBM patients, the elderly population was frequently excluded from these studies [38,39]. Additionally, preexisting comorbidities may influence neurosurgical decision making to withhold elderly patients from aggressive resections with the aim of GTR [40]. However, recent studies showed that complete GBM resection has the greatest benefit for survival among all age subgroups [41,42].

### 4.5. Safety of 5-ALA Procedures in Elderly Patients

In this study, 5-ALA administration did not result in any severe side effects and was well-tolerated by our elderly GBM cohort. In consideration of the high patient age, only three patients had to be excluded from 5-ALA administration due to markedly elevated liver enzymes or internal contraindications. Our findings in this elderly population are in agreement with two previous studies including HGG patients with a lower median age between 60 and 63 years reporting no toxicological safety issues regarding the 5-ALA application [8,43]. Additionally, one [8] of these studies analyzed different laboratory measurements which were mildly elevated 24 h after surgery [8]. However, these reported findings were transient, showed no clinical relevance, and may also have been related to anesthesiologic medication applied during surgery [8]. Other studies described only mild side effects after 5-ALA administration such as intraoperative hypotension in a very low portion of patients [44,45]. Further, the rate of symptomatic postsurgical hemorrhages and postoperative neurological deterioration was low in our study and comparable to the literature [46,47]. Thus, 5-ALA administration is safe in elderly patients and the benefits of intraoperative fluorescence visualization of GBM in resections as well as biopsies should not be restrained in this age group.

### 4.6. Limitations

(1) Firstly, our study design represents a retrospective analysis and is thus associated with its known shortcomings. (2) Secondly, we did not have available a meaningful control group without 5-ALA use since we applied this fluorescent dye routinely in resections as well as biopsies within previous clinical studies in most patients [14,16]. (3) Thirdly, not all tumors were classified according to the present WHO criteria from 2016 since tumor diagnosis was established in this study according to the current WHO classification at the time of diagnosis [1,20]. (4) Furthermore, different scores exist to assess the overall level of fitness/frailty of older adults including the “Frailty score” [48]. In the present study, we selected the Charleston index in our study since this score is well established at our center. (5) Moreover, we did not systematically collect data on the length of hospital stay and the exact time point of initiation of postoperative chemo-/radiotherapy in each patient. These parameters should be included in future studies in elderly cohorts. (6) In addition, we did not have available a meaningful elderly control group with GBM resection without preoperative 5-ALA administration since the 5-ALA technique is routinely applied in GBM surgery at our department and thus only a few patients (n = 34) did not receive 5-ALA during the study period. (7) Additionally, the outcome of a large elderly series with 5-ALA fluorescence-guided GBM resection should be compared with a younger cohort in future studies. (8) Further, we cannot make a statement on the diagnostic accuracy since we used the presence of WHO grade IV glioma tissue as inclusion criterion in this retrospective study to analyze the frequency of cases with visible fluorescence in this elderly cohort. However, it would be very interesting to compare the rate of fully diagnostic biopsies after 5-ALA administration in an elderly cohort in a future prospective study to an institutional historical cohort without 5-ALA application. (9) Finally, we have experience only with the fluorescence dye 5-ALA at our center. Nevertheless, other fluorescent dyes, such as sodium fluorescein, might have the potential to intraoperatively support the identification of GBM tissue in biopsies or increase the extent of resection in surgical removal in elderly patients [49,50].

## 5. Conclusions

In this largest series to date, we demonstrate the clinical utility and safety of 5-ALA fluorescence in elderly GBM patients for improved tumor visualization in both resections as well as biopsies. In this sense, tumor tissue was intraoperatively visualized by 5-ALA fluorescence in the vast majority of GBM (99%). In resections, a high rate of GTR was achieved in completely resectable tumors with assistance of 5-ALA fluorescence resulting in improved overall survival. In biopsies, diagnostic tumor tissue could be identified directly in the operating room, enabling the advantage to markedly shorten these procedures. Thus, we suggest this fluorescence strategy in elderly GBM patients with the use of 5-ALA not only in resections, but also in open/stereotactic biopsies to optimize neurosurgical management.

## Figures and Tables

**Table 1:** Patient characteristics.

		*N*	%
Number of patients		272	(100)
Gender	female:male	1:1.1	
Age	median (range)	71 years (65–88)	
Preoperative KPS			
	100%	9	(3)
	90%	52	(19)
	80%	74	(27)
	70%	44	(16)
	≤60%	93	(35)
Number of surgeries per patient			
	1 surgery	244	(90)
	2 surgeries	25	(9)
	≥3 surgeries	3	(1)
Histology	glioblastoma	269	(99)
	gliosarcoma	3	(1)
Charlson Comorbidity Index			
	2	33	(12)
	3	45	(16)
	4	56	(20)
	5	57	(20)
	6	53	(19)
	>7	28	(10)
Karnofsky performance status (KPS)			

**Table 2:** Surgery characteristics.

		*N*	*%*
Number of patients		272	(100)
Number of surgeries		289	(100)
Newly diagnosed vs. recurrent			
	newly diagnosed	264	(91)
	recurrent	25	(9)
Type of surgery			
	resection	182	(63)
	stereotactic biopsy	84	(29)
	open biopsy	23	(8)
Localization	temporal	86	(30)
	frontal	67	(23)
	parietal	38	(13)
	central	23	(8)
	corpus callosum	19	(6)
	occipital	15	(5)
	trigonal	14	(5)
	basal ganglia	11	(4)
	other	16	(6)
Multicentric/multifocal			
	single lesion	217	(75)
	multifocal	53	(18)
	multicentric	19	(7)
Eloquence	eloquent	163	(56)
	non-eloquent	126	(44)
CE on MRI	ring-like	254	(88)
	nodular	23	(8)
	patchy/faint	6	(2)
	focal	2	(1)
	no available MRI	4	(1)
5-ALA fluorescence-guided surgery			
	fluorescence guided	255	(88)
	conventional	34	(12)
Maximal fluorescence status			
	strong	236	(93)
	vague	16	(6)
	none	3	(1)
5-aminolevulinic acid (5-ALA), contrast-media enhancement (CE),			
magnetic resonance imaging (MRI)			

## Data Availability

The data presented in this study are available on request from the corresponding author.

## Supplementary Materials

The following are available online at https://www.mdpi.com/article/10.3390/cancers13236119/s1, Table S1: Charlson Comorbidity Index (CCI), Table S2: Reasons for no 5-ALA administration, Table S3: Postoperative treatment of newly diagnosed GBM WHO grade IV.
