# Supplementary material for: Efficacy, Outcome, and Safety of Elderly Patients with Glioblastoma in the 5-ALA Era: Single Center Experience of More Than 10 Years
Source: Cancers (Basel). 2021 Dec 4;13(23):6119. doi: 10.3390/cancers13236119 (PMC8657316; doi:10.3390/cancers13236119)
Supplement: Supplementary file 1 [file cancers-13-06119-s001.zip › cancers-1429406-SI.pdf]

# Supplementary Material: Efficacy, Outcome, and Safety of Elderly Patients with Glioblastoma in the 5-ALA Era: Single Center Experience of More than 10 Years

**Barbara Kiesel, Lisa Wadiura, Mario Mischkulnig, Jessica Makolli, Veronika Sperl, Martin Borkovec, Julia Freund, Alexandra Lang, Matthias Millesi, Anna S. Berghoff, Julia Furtner, Adelheid Woehrer, Georg Widhalm**

**Table S1.** Charlson Comorbidity Index (CCI).

| Disease | | Points |
|---|---|---|
| Myocardial infarction | | 1 |
| CHF Congestive heart failure | | 1 |
| Peripheral vascular disease | | 1 |
| CVA or TIA | | 1 |
| Dementia | | 1 |
| COPD | | 1 |
| Connective tissue disease | | 1 |
| Peptic ulcer disease | | 1 |
| Liver disease | mild* | 1 |
| | moderate⁑ to severe⁂ | 3 |
| Diabetes mellitus | none or diet-controlled | 0 |
| | uncomplicated | 1 |
| | end-organ damage | 2 |
| Hemiplegia | | 2 |
| Moderate† to severe CKD‡ | | 2 |
| Solid tumor | localized | 2 |
| | metastatic | 6 |
| Leukemia | | 2 |
| Lymphoma | | 2 |
| AIDS | | 6 |
| Age | <50 years | 0 |
| | 50–59 years | 1 |
| | 60–69 years | 2 |
| | 70–79 years | 3 |
| | ≥80 years | 4 |

*mild = chronic hepatitis (or cirrhosis without portal hypertension)
⁑moderate=cirrhosis and portal hypertension but no variceal bleeding history
⁂severe= cirrhoseis and portal hypertension with variceal bleeding history
†moderate = creatinen > 3 mg/dL
‡severe=on dialysis, status post kidney transplant, uremia

| *1-year-mortality rate* | |
|---|---|
| 0 point | 12% |
| 1–2 points | 26% |
| 3–4 points | 52% |
| >5 points | 85% |

*Estimated 10-year survival* = $0.983\^{}(e^{CCI \times 0.9})$

Charlson comorbidity index (CCI), chronic kidney disease (CKD), chronic obstructive pulmonary disease (COPD), cerebrovascular accident (CVA), transient ischemic attack (TIA).

**Table S2.** Reasons for no 5-ALA administration.

| | ***n*** | **%** |
|---|---|---|
| Patients with 5-ALA administration | 255 | (88) |
| Patients without 5-ALA administration | 34 | (12) |
| other tumor entity | 9 | (26) |
| stereotactic biopsy* | 6 | (17) |
| inadequate administration | 5 | (15) |
| emergency surgery | 4 | (12) |
| preference of the surgeon | 3 | (9) |
| elevated liver enzymes | 2 | (6) |
| incapable to give informed consent | 2 | (6) |
| endoscopic procedure | 1 | (3) |
| internal contraindications | 1 | (3) |
| declined informed consent | 1 | (3) |

5-aminolevulinic acid (5-ALA). *Study "5-ALA in stereotactic biopsies" was not initiated at this timepoint.

**Table S3.** Postoperative treatment of newly diagnosed GBM WHO grade IV.

| | *n* | % |
|---|---|---|
| Patients without postoperative treatment | 63 | (24) |
| Patients with postoperative treatment | 201 | (76) |
| | | |
| RTX (60Gy) + TMZ | 88 | (43.8) |
| RTX (34Gy) + TMZ | 63 | (31.3) |
| RTX (40.05Gy) + TMZ | 17 | (8.5) |
| RTX (34Gy) alone | 13 | (6.5) |
| RTX (60Gy) alone | 10 | (5.0) |
| TMZ alone | 4 | (2.0) |
| RTX (49Gy) alone | 2 | (1.0) |
| RTX (19Gy) + TMZ | 1 | (0.5) |
| Dabrafenib + Trametinib | 1 | (0.5) |
| RTX + TMZ + investigative drug | 1 | (0.5) |
| RTX (40.05Gy) alone | 1 | (0.5) |

Gray (Gy), radiotherapy (RTX), temozolomide (TMZ).
